# Evaluation of Circulating Levels of ICAM-1 in Obstructive Sleep Apnea (OSA) Adults: Systematic Review, Meta-Analysis, and Trial Sequential Analysis of Link Between OSA and Cardiovascular Disease

**DOI:** 10.3390/life15081278

**Published:** 2025-08-12

**Authors:** Mohammad Moslem Imani, Arya Imani, Masoud Sadeghi, Annette Beatrix Brühl, Serge Brand

**Affiliations:** 1 Department of Orthodontics, Kermanshah University of Medical Sciences, Kermanshah 6713954658, Iran; mmoslem.imani@yahoo.com; 2 Students Research Committee, Kermanshah University of Medical Sciences, Kermanshah 6715847141, Iran; arya.imani@kums.ac.ir; 3 Medical Biology Research Center, Health Technology Institute, Kermanshah University of Medical Sciences, Kermanshah 6714415185, Iran; sadeghi_mbrc@yahoo.com; 4 Center for Affective, Stress and Sleep Disorders, Psychiatric Clinics, University of Basel, 4002 Basel, Switzerland; annette.bruehl@upk.ch; 5 Sleep Disorders Research Center, Kermanshah University of Medical Sciences, Kermanshah 6715847141, Iran; 6 Substance Abuse Prevention Research Center, Kermanshah University of Medical Sciences, Kermanshah 6715847141, Iran; 7 Division of Sport Science and Psychosocial Health, Department of Sport, Exercise and Health, University of Basel, 4002 Basel, Switzerland; 8 School of Medicine, Tehran University of Medical Sciences, Tehran 1416753955, Iran; 9 Center for Disaster Psychiatry and Disaster Psychology, Center of Competence for Disaster Medicine, Swiss Armed Forces, 4002 Basel, Switzerland

**Keywords:** obstructive sleep apnea, intercellular adhesion molecule-1, cardiovascular diseases, biomarkers, meta-analysis

## Abstract

Obstructive sleep apnea (OSA) is a common condition characterized by repeated airway collapses during sleep, contributing to oxygen desaturation, arousals, and significant cardiovascular complications. This meta-analysis aims to evaluate the association between blood ICAM-1 levels and OSA, exploring its potential as a biomarker for cardiovascular disease (CVD) and for identifying factors contributing to result heterogeneity. Following PRISMA guidelines, this meta-analysis addressed a PECO framework to assess circulating ICAM-1 levels in adults with OSA compared to controls. A systematic search was conducted across PubMed, Web of Science, Scopus, Cochrane Library, and CNKI until 23 April 2025, complemented by citation reviews and Google Scholar. Statistical analyses, including subgroup and meta-regression, were performed using RevMan, CMA 3.0, and TSA software to calculate mean differences, assess heterogeneity, and evaluate publication bias. Results were analyzed under random-effect models, with significance set at *p* < 0.05 for all metrics except publication bias (*p* < 0.10). This systematic review and meta-analysis included 34 articles. The pooled mean difference (MD) of ICAM-1 levels was 184.06 ng/mL (95% CI: 143.83 to 224.28; *p* < 0.00001), significantly higher in OSA patients with high heterogeneity (I^2^ = 100%). Subgroup analysis highlighted larger MDs in Asians and plasma samples, as well as greater ICAM-1 elevations in severe OSA cases. Despite publication bias indicated by Begg’s (*p* = 0.036) and Egger’s (*p* = 0.016) tests, the findings remained robust, supported by sensitivity and meta-regression analyses. This meta-analysis underscores a significant association between elevated ICAM-1 levels and OSA, highlighting its potential as a biomarker for CVD risk stratification in OSA patients.

## 1. Introduction

Obstructive sleep apnea (OSA) is a condition characterized by the repeated collapse of the upper airway during sleep, resulting in episodes of oxygen desaturation and frequent arousals [1,2,3,4]. Among young adults, the prevalence of OSA is estimated to be approximately 16%, although variations in hypopnea definition, apnea–hypopnea index (AHI) thresholds, and device types have contributed to inconsistencies in prevalence across studies [5]. The AHI, defined as the number of apneas and hypopneas per hour of sleep, is commonly used to diagnose OSA in adults, with a threshold of five or more events per hour indicating its presence [6,7,8]. This index also categorizes disease severity: individuals with an AHI of 5–15, 16–30, or over 30 events per hour are classified as having mild, moderate, or severe OSA, respectively [6,9,10].

OSA has been increasingly implicated in several cardiovascular complications, including stroke, transient ischemic attacks, coronary heart disease, heart failure, cardiac arrhythmias, and pulmonary hypertension [11,12,13,14]. While the precise mechanisms underlying these associations remain unclear, hypoxia induced by OSA plays a significant role in activating adhesion molecules such as intercellular adhesion molecule-1 (ICAM-1; CD54), which contributes to vascular inflammation and dysfunction [15]. Risk factors for OSA include obesity, craniofacial or oropharyngeal anatomical abnormalities, male sex, and smoking [16]. Moreover, severe OSA has been identified as an independent predictor of all-cause and cardiovascular mortality, irrespective of race or ethnicity [17].

ICAM-1, a 90 kDa protein from the immunoglobulin (Ig) superfamily, plays a pivotal role in leukocyte arrest and transmigration across blood vessels into tissues [18,19]. Plasma ICAM-1 levels have been linked to an increased risk of myocardial infarction, coronary death, and angina pectoris [20,21]. Elevated baseline concentrations of ICAM-1 are also associated with a higher likelihood of developing macrovascular disease [22]. Soluble ICAM-1 levels reflect established cardiovascular disease (CVD) risk factors in apparently healthy individuals, highlighting the role of vascular inflammation in disease progression [23]. Additionally, ICAM-1 has been implicated in vascular dysfunction and hypertension driven by Angiotensin II [24].

Notably, significantly higher levels of ICAM-1 have been observed in individuals with OSA compared to non-OSA counterparts, further supporting its role as a critical mediator of OSA-induced CVD risks [15]. OSA can independently elevate circulating levels of adhesion molecules, such as ICAM-1 [25]. In non-OSA populations, increased ICAM-1 levels are associated with a 5.53-fold higher risk of incident coronary heart disease [26].

The aim of this meta-analysis is to provide a comprehensive and updated evaluation of the association between blood levels of ICAM-1 and OSA while exploring the broader implications of ICAM-1 in CVD among OSA patients. Building on previous meta-analyses involving 8 and 17 articles, respectively [27,28], this study incorporates data from 34 newly published articles, addresses existing limitations, and examines additional variables, including ethnicity, disease severity, and sample type. By employing rigorous subgroup and meta-regression analyses, this work seeks to enhance the understanding of ICAM-1’s clinical significance as a biomarker for OSA and CVD risk while identifying factors contributing to heterogeneity in results.

## 2. Materials and Methods

This meta-analysis followed PRISMA guidelines [29] and addressed a PECO question to examine circulating levels of ICAM-1 in adults with OSA compared to controls. The PECO framework was constructed as follows: Population (P) consisted of adults diagnosed with OSA; Exposure (E) referred to the presence of OSA; Comparator (C) included adults without OSA (controls); and Outcome (O) focused on circulating levels of ICAM-1. The systematic review and meta-analysis were registered in the PROSPERO database (ID: CRD420251043761).

### 2.1. Study Selection

A systematic literature search was conducted across multiple databases, including PubMed, Web of Science, Scopus, Cochrane Library, and CNKI, until 23 April 2025, without any restrictions by one author (M.S.). The search strategy utilized in the databases included the following terms: (“OSAHS” or “OSA” or “OSAS” or “sleep apnea” or “obstructive sleep apnea” or “obstructive sleep apnea syndrome” or “obstructive sleep apnea-hypopnea syndrome” or “obstructive sleep apnoea/hypopnoea syndrome”) and (“intercellular adhesion protein” or “Intercellular adhesion molecule” or “ICAM*” OR “CD54” OR “cluster of differentiation 54”) and (“serum” or “plasma” or “blood” or “circulating”). After searching among the databases, we removed duplicates and then checked the title/abstract of each article based on the eligibility criteria. We also reviewed the citations of relevant reviews and meta-analyses related to the subject and utilized Google Scholar to identify any potentially missing articles. Another author (M.M.I.) rechecked all processes of study selection, and any disagreements between the two authors were resolved by the third author (S.B.).

### 2.2. Eligibility Criteria

The inclusion criteria encompassed studies involving adults of any type that included both an OSA case group and a control group. OSA was defined as an AHI ≥ 5 events/h, while controls were classified with an AHI less than 5 events/h. Studies were required to report serum or plasma levels of ICAM-1. Both the OSA group and the control group were stipulated to be free from systemic diseases. OSA was diagnosed by polysomnography.

The exclusion criteria ruled out studies with fewer than ten cases in one or both groups, articles with incomplete or missing data, reviews, meta-analyses, and duplicate publications.

### 2.3. Quality Score

We used the Newcastle–Ottawa Scale (NOS) questionnaire [30] to assess study quality, with a maximum score of 9. The first section, Selection, could score up to 4 points, focusing on the quality of group selection and representativeness. The second section, Comparability, allowed a maximum of 2 points, assessing how well confounding factors were controlled. The third section, Outcome or Exposure, had a maximum of 3 points, evaluating the methods of measurement and adequacy of follow-up or response rates. Studies scoring ≥ 7 were considered high quality.

### 2.4. Radial Plot

We utilized the NCSS 2021 software to generate a radial plot, an effective graphical method for visualizing heterogeneity in meta-analyses [31]. A radial plot is particularly useful in detecting outliers that may contribute to heterogeneity within the data. By plotting the study weights against their standardized effect sizes, this approach enables a quick identification of studies deviating significantly from the overall trend, ensuring a clearer understanding of their impact on the meta-analysis results.

### 2.5. Statistical Analyses

For the analyses conducted in the study, we utilized various software tools to ensure accuracy and robustness in our findings. The Review Manager (RevMan) 5.1 software was employed to calculate mean differences (MDs) and 95% confidence intervals (CI) in funnel plots, enabling the visualization of potential asymmetry and identification of publication bias. Comprehensive Meta-Analysis (CMA) 3.0 software was used for sensitivity analyses, meta-regression analyses, and assessment of publication bias. These analyses allowed us to evaluate the stability of results, explore potential moderators, and examine the impact of publication bias on the meta-analysis outcomes. Additionally, Trial Sequential Analysis (TSA) software (version 0.9.5.10 beta) was used to perform TSA analyses [32,33], which assessed the robustness and reliability of the results by accounting for random error and ensuring that sufficient evidence was accumulated for definitive conclusions. In conducting the TSA, we used an alpha level of 5% and a beta of 80%, adhering to standard statistical conventions for controlling Type I and Type II errors [34,35]. The MD was calculated empirically within the TSA framework to ensure precise assessment of the cumulative evidence while accounting for random errors.

A *p*-value of less than 0.05 was considered statistically significant for all analyses, except for the assessment of publication bias, where a threshold of *p* < 0.10 [36,37] was used. Additionally, random-effect analyses [38] were employed, as the heterogeneity among studies, indicated by an I^2^ value greater than 50%, warranted this approach to account for variability across the included studies.

## 3. Results

### 3.1. Study Selection Process Summary

**Figure 1:**
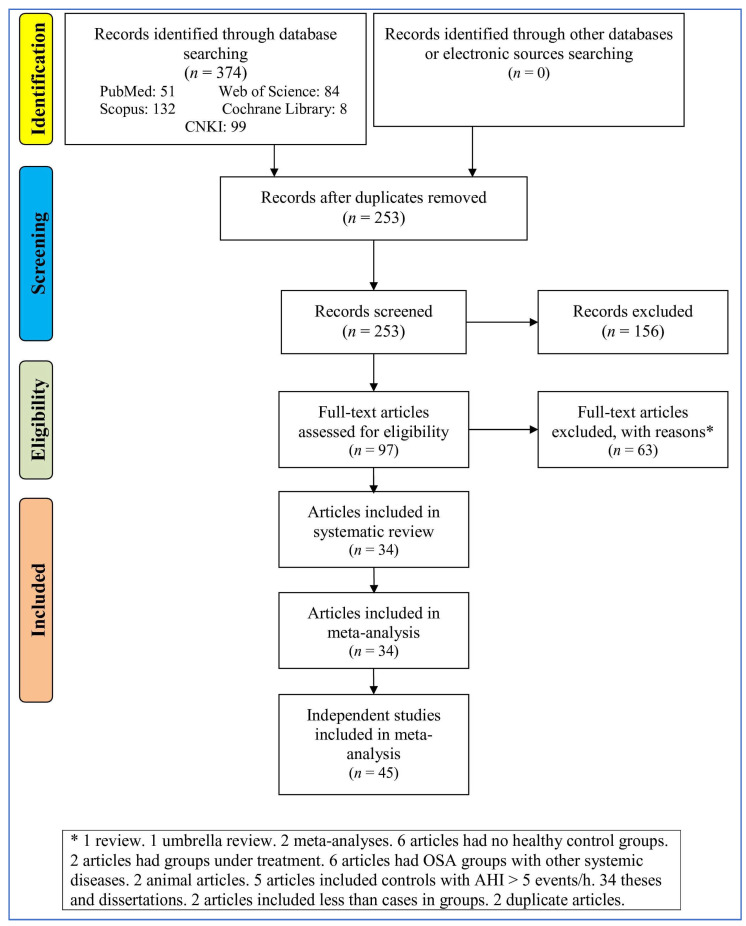
Flowchart of study selection.

Figure 1 outlines the selection process for a systematic review and meta-analysis. Initially, 374 records were identified across databases, narrowed to 253 after duplicate removal. Screening excluded 156 records, and 97 full-text articles were assessed for eligibility, with 63 excluded for reasons such as lacking healthy controls or involving animal studies. Ultimately, thirty-four articles [25,39,40,41,42,43,44,45,46,47,48,49,50,51,52,53,54,55,56,57,58,59,60,61,62,63,64,65,66,67,68,69,70,71] were included in the meta-analysis; one article [60] included two independent studies (mild and moderate/severe cases vs. controls) and five articles [44,62,63,64,65] included three independent studies (mild, moderate, and severe cases vs. controls). Therefore, 45 independent studies were entered into the analysis.

### 3.2. Characteristics of Articles Included in the Meta-Analysis

Table 1 presents key details of the articles incorporated into the meta-analysis. These articles span diverse ethnicities, including Caucasian, Asian, and mixed populations, with varying sample types (serum or plasma). The age, body mass index (BMI), and AHI data for cases and controls provide insights into the study populations, and the Newcastle–Ottawa Scale (NOS) scores, ranging from 5 to 9, reflect the quality of the articles.

### 3.3. Blood Levels of ICAM-1 in Cases and Controls

Table 2 compares the blood levels of ICAM-1 between cases and controls across various articles. It includes sample sizes for each group and reports mean levels with standard deviations (mean ± SD). The data demonstrate notable differences in ICAM-1 levels between cases and controls, highlighting trends where higher ICAM-1 levels are often observed in cases compared to controls, which may indicate its role in disease processes.

### 3.4. Forest Plot Analysis of ICAM-1 Blood Levels in Cases vs. Controls

**Figure 2:**
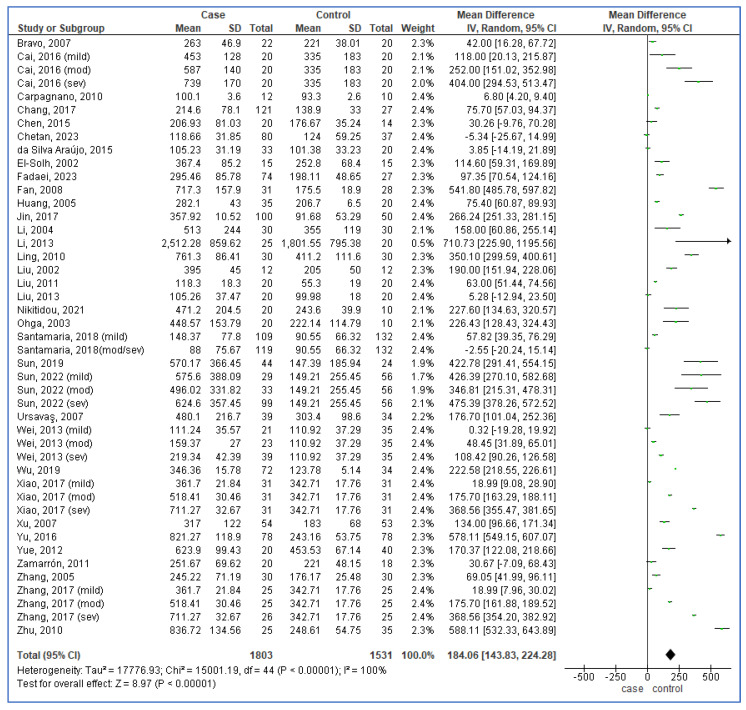
Forest plot analysis of blood levels of intercellular adhesion molecule-1 in cases compared to controls. Each row represents a study, detailing its mean, standard deviation (SD), and sample size for both groups. The mean difference and 95% confidence interval (CI) for each study are also plotted. Squares represent individual studies, with their size reflecting study weight in the meta-analysis, while horizontal lines depict the CI. The diamond at the bottom summarizes the overall effect size, emphasizing the aggregated results [25,39,40,41,42,43,44,45,46,47,48,49,50,51,52,53,54,55,56,57,58,59,60,61,62,63,64,65,66,67,68,69,70,71].

A random-effect forest plot, shown in Figure 2, illustrates the comparison of blood levels of ICAM-1 between cases and controls across multiple studies. The pooled MD of ICAM-1 levels was calculated as 184.06 ng/mL, indicating higher levels in cases compared to controls. The 95% CI—143.83 to 224.28—shows the range within which the true MD is expected to lie with high certainty. The *p*-value < 0.00001 signifies a statistically significant result, meaning the observed difference is unlikely due to chance. Lastly, I^2^ = 100% indicates high heterogeneity among the included studies, reflecting variability in results across the studies. This analysis underscores the potential association of elevated ICAM-1 levels with the investigated condition.

### 3.5. Radial Plot Analysis of ICAM-1 Blood Levels in Cases vs. Controls

**Figure 3:**
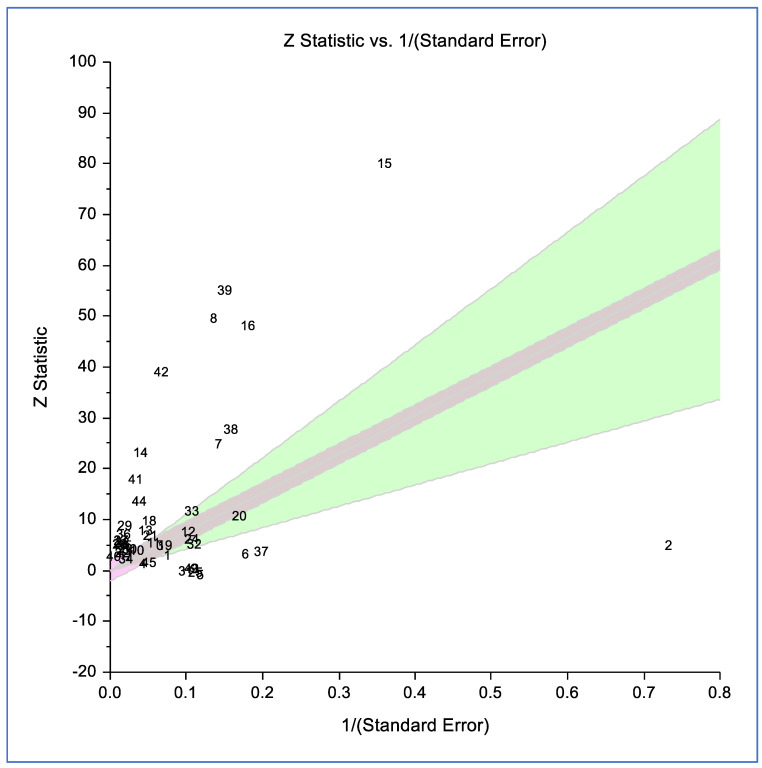
Radial plot of blood levels of intercellular adhesion molecule-1 in cases compared to controls. The plot visualizes individual data points, showing the relationship between “Z statistic” and “1/(standard error).” The shaded region represents a confidence interval or range, and the significant *p*-value (<0.001) highlights the statistical difference between the groups.

Figure 3 presents a radial plot comparing the blood levels of intercellular adhesion molecule-1 (ICAM-1) between cases and controls. High heterogeneity, indicated by a significant *p*-value < 0.001 and possibly supported by an I^2^ statistic, suggests considerable variability in the data across studies. Outliers could influence the overall results, potentially affecting the robustness of the pooled analysis. Removing potential outliers, heterogeneity did not reduce; it indicated that substantial heterogeneity still exists, even after adjustments.

### 3.6. Trial Sequential Analysis of ICAM-1 Blood Levels in Cases vs. Controls

**Figure 4:**
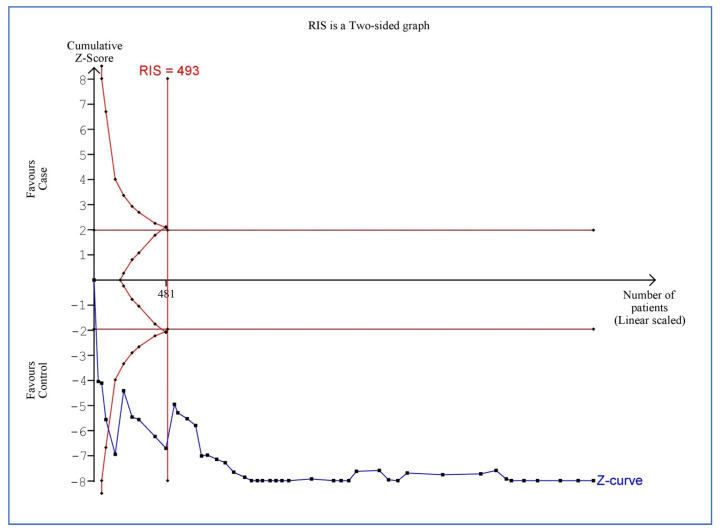
Trial sequential analysis of blood levels of intercellular adhesion molecule-1 in cases compared to controls.

Figure 4 showcases the TSA comparing blood levels of ICAM-1 between cases and controls. The graph evaluates cumulative evidence as the sample size increases, ensuring the reliability of the meta-analysis findings. The cumulative Z-curve (blue) tracks the progression of significance, while boundaries indicate the RIS. The Z-curve crosses these boundaries; the effect is statistically significant, and further trials may not be required.

### 3.7. Stability of Pooled Data in ICAM-1 Analysis

The cumulative and one-study-removed analyses demonstrated consistent results, confirming the robustness of the pooled mean difference for blood levels of ICAM-1 in cases compared to controls. In cumulative analysis, the inclusion of additional studies did not alter the pooled data significantly, indicating stable and reliable findings as sample size increased. Similarly, the one-study-removed analysis, which evaluates the impact of individual studies on overall results, showed no meaningful changes in the pooled data when any single study was excluded. This stability underscores the strength of the meta-analysis and the reliability of its conclusions.

Initially, the pooled MD was 184.06 ng/mL (I^2^ = 100%, *p* < 0.00001), indicating significant heterogeneity and a strong association. After excluding articles with a quality score of less than 7 [45,50,57,60,62], the recalculated pooled MD slightly decreased to 172.39 ng/mL (I^2^ = 100%, *p* < 0.00001), maintaining statistical significance. This suggests that the exclusion of lower-quality studies had minimal impact on the overall findings, reinforcing the reliability of the meta-analysis results, and therefore, the main cause of heterogeneity is not the quality of the studies.

### 3.8. Subgroup Analysis

Subgroup analysis reveals significant variations in ICAM-1 levels between OSA cases and controls across different variables (Table 3). Ethnicity shows the largest MD in Asians (223.53 ng/mL, *p* < 0.00001, I^2^ = 100%), followed by Caucasians (50.66 ng/mL, *p* = 0.0001, I^2^ = 93%), while mixed ethnicity results are not statistically significant (*p* = 0.31). Sample size does not affect outcomes, as studies with both ≥100 participants (186.38 ng/mL) and <100 participants (181.88 ng/mL) exhibit significant results (*p* < 0.00001, I^2^ = 100%). Higher apnea–hypopnea index (AHI) values (≥30 events/h) lead to greater MDs (180.97 ng/mL) compared to lower AHI values (<30 events/h; 135.54 ng/mL). Blood sample type also plays a role, with plasma showing a higher MD (238.53 ng/mL) than serum (169.29 ng/mL). Significant *p*-values across most subgroups underline consistency, but high heterogeneity persists (I^2^ ≥ 99%).

### 3.9. Meta-Regression Analysis

The meta-regression analysis identifies factors influencing ICAM-1 blood level differences between OSA cases and controls (Table 4). Among the variables analyzed, mean AHI in cases shows a statistically significant association (coefficient = 3.6421, *p* = 0.0213), indicating that higher AHI values in cases correlate with increased ICAM-1 levels. Conversely, publication year and sample size did not exhibit statistically significant effects on ICAM-1 levels, with *p*-values of 0.9826 and 0.4679, respectively. This suggests that disease severity (as measured by AHI) plays a more critical role than publication timing or study size in explaining differences in ICAM-1 levels.

### 3.10. Publication Bias

**Figure 5:**
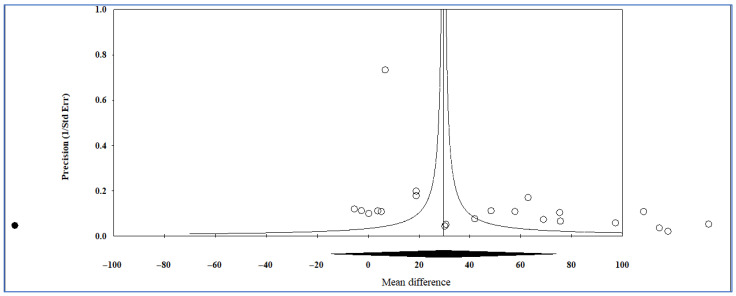
Funnel plot of blood levels of intercellular adhesion molecule-1 in cases compared to controls. The plot shows the distribution of observed studies (○) and imputed studies (●) identified by the trim-and-fill method to assess potential publication bias. The x-axis represents the mean difference (effect size), and the y-axis indicates study precision (1/Standard Error). The vertical line denotes the adjusted overall effect size after accounting for missing studies. The asymmetry of the funnel and the increase in Q value after adjustment further support this interpretation.

Begg’s test (*p* = 0.036) and Egger’s test (*p* = 0.016) both indicate evidence of publication bias, as their *p*-values fall below the threshold of 0.10. This suggests that small studies with ‘null’ or ‘unfavorable’ results may have been underreported. The consistent findings from both tests further support the presence of bias, which could influence the robustness and validity of the pooled result. Figure 5 shows the funnel plot of blood levels of ICAM-1 with the trim-and-fill method in cases compared to controls.

Table 5 shows the results of a trim-and-fill analysis assessing the impact of publication bias on the meta-analysis estimates. The observed effect sizes (before adjustment) are compared to the adjusted estimates (after imputing potentially missing studies). Under the random-effect model, the observed point estimate was 183.976 with a 95%CI of 143.371 to 224.579, while the adjusted estimate decreased substantially to 29.675 (95% CI: –14.561 to 73.912), indicating that publication bias may have inflated the apparent effect. The Q value (13268.783) reflects significant heterogeneity among included studies. The fixed-effects model showed a similar pattern of reduction, with the point estimate dropping from 76.427 to 15.741 after adjusting for 22 trimmed studies.

## 4. Discussion

OSA should be recognized as a multifactorial condition influenced by various genetic, environmental, and developmental factors [72]. This meta-analysis highlights a significant association between elevated blood levels of ICAM-1 and OSA, with a pooled MD of 184.06 ng/mL, reaffirming its statistical significance (*p* < 0.00001).

Despite robust findings validated through sensitivity analyses, high heterogeneity (I^2^ = 100%) persists, likely due to variations in ethnicity and OSA severity. In addition to ethnicity and OSA severity, the extremely high heterogeneity (I^2^ = 100%) observed may stem from several other factors. These include differences in diagnostic criteria for OSA across studies (e.g., varying AHI cut-offs), lack of standardization in ICAM-1 measurement techniques (e.g., different assay platforms or sample types such as serum vs. plasma), as well as variations in study population age, sex distribution, comorbidities, and lifestyle-related variables such as smoking status, physical activity, and body mass index. These methodological and clinical inconsistencies limit the direct comparability of the included studies and reduce the generalizability of the pooled estimate. Consequently, while the association between elevated ICAM-1 levels and OSA remains statistically significant, these findings should be interpreted with caution. Future studies employing standardized diagnostic and biomarker assessment protocols across diverse populations are needed to confirm and clarify the strength of this association.

Our trim-and-fill analysis revealed a notable difference between observed and adjusted effect sizes, suggesting potential publication bias. After accounting for 22 missing studies, the effect estimate decreased and lost statistical significance, indicating that the original findings may have been overestimated. This highlights the need for cautious interpretation and underscores the importance of including unpublished or negative-result studies in future analyses to reduce bias.

Subgroup and meta-regression analyses emphasize the role of disease severity, particularly higher AHI values, in driving ICAM-1 elevation. However, evidence of publication bias and methodological differences across studies necessitates cautious interpretation of the results.

OSA is notably prevalent, affecting an estimated 34% of men and 17% of women in the general population, as well as 40% to 60% of individuals with CVD [16]. Despite its high prevalence, OSA remains underdiagnosed, particularly among patients with cardiovascular conditions. The presence of OSA significantly elevates the risk of cardiovascular mortality and morbidity, being closely linked to resistant hypertension, heart failure, arrhythmias, and coronary artery disease [73]. Importantly, the treatment of OSA has been shown to effectively reduce the incidence and prevalence of these CVDs [74].

In a cohort of suspected OSA patients, those with elevated ICAM-1 levels (>816 ng/mL) were significantly more likely to experience a cardiovascular event within 8 years following polysomnography [75]. ICAM-1 facilitates the binding and recruitment of white blood cells to the endothelium [76], contributes to the formation of atherosclerotic plaques, and is associated with the development of CVD [26,77,78]. The evidence also underscores the role of disease severity, especially higher AHI values, in driving ICAM-1 elevation, which further links OSA to cardiovascular pathology. While larger validation studies are necessary, ICAM-1 shows potential as a biomarker to identify OSA patients at heightened risk of future cardiovascular events [75].

In summary, given the strong association between elevated ICAM-1 levels and cardiovascular events in OSA patients, ICAM-1 could serve as a valuable biomarker for identifying individuals at higher risk of adverse outcomes. Incorporating ICAM-1 measurements into routine clinical assessments may enhance risk stratification and guide personalized therapeutic interventions. Furthermore, targeting ICAM-1 pathways through pharmacological or lifestyle modifications could potentially mitigate CVD risks in OSA patients. Future research should focus on validating these findings in larger cohorts and exploring the mechanistic links between ICAM-1 and cardiovascular pathology in OSA.

### Limitations

1. High Heterogeneity: Despite various adjustments, heterogeneity (I^2^ = 100%) remains significant, indicating substantial variability across studies, which may stem from differences in methodology, populations, or study designs.
2. Publication Bias: Evidence of publication bias, as suggested by Begg’s (*p* = 0.036) and Egger’s (*p* = 0.016) tests, points to the underrepresentation of smaller studies with null results, potentially influencing the pooled outcomes.
3. Data Variability: Differences in demographic factors (e.g., ethnicity, AHI severity, blood sample type) create challenges in drawing universally applicable conclusions.

## 5. Conclusions

With regard to high heterogeneity, the meta-analysis reveals a strong association between elevated ICAM-1 blood levels and OSA. Robustness is evident through sensitivity analyses, while subgroup analysis highlighted ethnicity, and meta-regression showed AHI of OSA cases as influential factors.

Elevated ICAM-1 levels in OSA patients may serve as a potential biomarker for disease severity, particularly in those with higher AHI values. This insight could aid in early detection, risk stratification, and tailored therapeutic strategies for OSA management, emphasizing the importance of monitoring inflammatory markers.

Future studies should focus on addressing heterogeneity by standardizing methodologies, exploring ethnic and biological variations, and ensuring comprehensive representation across diverse populations. Additionally, mechanisms underlying ICAM-1 elevation in OSA need further investigation to elucidate its clinical and pathophysiological roles. Incorporating multi-center studies and minimizing publication bias would enhance the reliability of future meta-analytic findings.

## Figures and Tables

**Table 1:** Characteristics of articles included in the meta-analysis.

First Author, Publication Year	Ethnicity	Case, Mean			Control, Mean			Sample	NOS Score
		Age, yrs	BMI, kg/m^2^	AHI, Events/h	Age, yrs	BMI, kg/m^2^	AHI, Events/h		
Bravo, 2007 [39]	Caucasian	52.3	30.9	48.9	47.4	28.4	2.5	Serum	8
Carpagnano, 2010 [40]	Caucasian	47.3	42.6	48.8	45.9	34.5	3.9	Plasma	7
Chang, 2017 [41]	Asian	43.8	25.1	35.6	39.9	24.4	2.4	Serum	9
Chen, 2015 [42]	Asian	38.6	27.13	58.89	38.21	23.84	2.86	Serum	9
Chetan, 2023 [43]	Caucasian	60	34	28.6	55	29	2.8	Serum	7
Zhang, 2017 [44]	Asian	70.1	25.31	11.07	68.7	24.92	1.75	Serum	7
		68.9	25.12	27.74					
		70.3	29.53	58.83					
da Silva Araújo, 2015 [45]	Mixed	39.6	34.39	20.16	32.5	34.54	2.55	Serum	6
El-Solh, 2002 [46]	Mixed	61.2	31.47	39.91	59.3	29.02	3.93	Plasma	9
Fadaei, 2023 [47]	Caucasian	45.97	26.7	18.9	45.63	26.1	2.25	Serum	9
Huang, 2005 [48]	Asian	51.0	-	≥5	49.0	-	<5	Plasma	7
Yue, 2012 [49]	Asian	44.36	26.8	26.20	45.50	27.80	3.20	Serum	9
Zhu, 2010 [50]	Asian	>18	-	≥5	>18	-	<5	Serum	5
Wu, 2019 [51]	Asian	54.9	25.8	35.6	53.6	23.6	3.5	Serum	8
Jin, 2017 [52]	Asian	55.28	26.74	38.01	56.13	25.19	3.62	Plasma	9
Li, 2004 [53]	Asian	41	29	53	45	26.9	<5	Serum	7
Liu, 2002 [54]	Asian	51.6	27.1	32.7	52.2	26.8	2.2	Serum	9
Zhang, 2005 [55]	Asian	67	28	≥5	68	26	<5	Serum	7
Liu, 2011 [56]	Asian	41.2	28.3	48.8	43.5	26.1	<5	Serum	8
Xu, 2007 [57]	Asian	>18	-	32	>18	-	2.1	Serum	6
Nikitidou, 2021 [58]	Caucasian	44.2	30.8	48.4	40.2	25.3	3.6	Serum	8
Ohga, 2003 [59]	Asian	47.8	29.4	38.9	48.9	28.4	3.1	Serum	9
Santamaria-Martos, 2018 [60]	Caucasian	57.67	28.07	9.33	44.4	24.87	1.89	Serum	6
		65.17	28.67	28.6					
Sun, 2019 [61]	Asian	43.84	27.59	57.57	44.8	23.42	2.62	Serum	7
Sun, 2022 [62]	Asian	47.5	25.13	5–15					
		48	26.67	15–30					
		45	29.03	>30	48	23.67	<5	Plasma	6
Ursavaş, 2007 [25]	Caucasian	52	30.8	50.5	49	28.8	1.9	Serum	8
Li, 2013 [63]	Asian	48.27	34.32	5–15	46.13	33.83	<5	Serum	7
		47.44	32.24	15–30					
		45.74	33.51	>30					
Cai, 2016 [64]	Asian	47	27.9	10.8	46	26.5	2.1	Plasma	9
		44	27.4	29.5					
		43	27.6	64					
Xiao, 2017 [65]	Asian	70.1	25.31	11.07	68.7	24.92	1.75	Serum	8
		68.9	25.12	27.74					
		70.3	29.53	58.83					
Li, 2013 [66]	Asian	47.08	29.30	≥5	44.05	25.07	<5	Serum	7
Fan, 2008 [67]	Asian	47.6	27.1	≥5	50.3	25.0	<5	Serum	7
Yu, 2016 [68]	Asian	60.5	25.32	28.53	58.2	23.45	1.48	Serum	8
Liu, 2013 [69]	Asian	45.80	26.99	40.87	48.30	27.33	3.05	Serum	9
Ling, 2010 [70]	Asian	47.6	-	≥5	47.6	-	<5	Serum	7
Zamarrón, 2011 [71]	Caucasian	50.1	29.9	45.2	44.1	27.6	<5	Serum	8

**Table 2:** Blood levels of intercellular adhesion molecule-1 in cases and controls.

First Author, Publication Year	Case (Number)	Control (Number)	Case (Mean ± SD), ng/mL	Control (Mean ± SD), ng/mL
Bravo, 2007 [39]	22	20	263.0 ± 46.9	221.0 ± 38.01
Carpagnano, 2010 [40]	12	10	100.1 ± 3.6	93.3 ± 2.6
Chang, 2017 [41]	121	27	214.6 ± 78.1	138.9 ± 33.0
Chen, 2015 [42]	20	14	206.93 ± 81.03	176.67 ± 35.24
Chetan, 2023 [43]	80	37	118.66 ± 31.85	124 ± 59.25
Zhang, 2017 [44]	Mild: 25 Mod: 25 Sev: 26	25	361.7 ± 21.84 518.41 ± 30.46 711.27 ± 32.67	342.71 ± 17.76
da Silva Araújo, 2015 [45]	33	20	105.23 ± 31.19	101.38 ± 33.23
El-Solh, 2002 [46]	15	15	367.4 ± 85.2	252.8 ± 68.4
Fadaei, 2023 [47]	74	27	295.46 ± 85.78	198.11 ± 48.65
Huang, 2005 [48]	35	20	282.1 ± 43.0	206.7 ± 6.5
Yue, 2012 [49]	20	40	623.90 ± 99.43	453.53 ± 67.14
Zhu, 2010 [50]	25	35	836.72 ± 134.56	248.61 ± 54.75
Wu, 2019 [51]	72	34	346.36 ± 15.78	123.78 ± 5.14
Jin, 2017 [52]	100	50	357.92 ± 10.52	91.68 ± 53.29
Li, 2004 [53]	30	30	513 ± 244	355 ± 119
Liu, 2002 [54]	12	12	395 ± 45	205 ± 50
Zhang, 2005 [55]	30	30	245.22 ± 71.19	176.17 ± 25.48
Liu, 2011 [56]	20	20	118.3 ± 18.3	55.3 ± 19.0
Xu, 2007 [57]	54	53	317 ± 122	183 ± 68
Nikitidou, 2021 [58]	20	10	471.2 ± 204.5	243.6 ± 39.9
Ohga, 2003 [59]	20	10	448.57 ± 153.79	222.14 ± 114.79
Santamaria-Martos, 2018 [60]	Mild: 109 Mod-sev: 119	132	148.37 ± 77.8 88.0 ± 75.67	90.55 ± 66.32
Sun, 2019 [61]	44	24	570.17 ± 366.45	147.39 ± 185.94
Sun, 2022 [62]	Mild: 29 Mod: 33 Sev: 99	56	575.6 ± 388.09 496.02 ± 331.82 624.6 ± 357.45	149.21 ± 255.45
Ursavaş, 2007 [25]	39	34	480.1 ± 216.7	303.4 ± 98.6
Li, 2013 [63]	Mild: 21 Mod: 23 Sev: 39	35	111.24 ± 35.57 159.37 ± 27.00 219.34 ± 42.39	110.92 ± 37.29
Cai, 2016 [64]	Mild: 20 Mod: 20 Sev: 20	20	453 ± 128 587 ± 140 739 ± 170	335 ± 183
Xiao, 2017 [65]	Mild: 31 Mod: 31 Sev: 31	31	361.70 ± 21.84 518.41 ± 30.46 711.27 ± 32.67	342.71 ± 17.76
Li, 2013 [66]	25	20	2512.28 ± 859.62	1801.55 ± 795.38
Fan, 2008 [67]	31	28	717.3 ± 157.9	175.5 ± 18.9
Yu, 2016 [68]	78	78	821.27 ± 118.90	243.16 ± 53.75
Liu, 2013 [69]	20	20	105.26 ± 37.47	99.98 ± 18.78
Ling, 2010 [70]	30	30	761.30 ± 86.41	411.20 ± 111.60
Zamarrón, 2011 [71]	20	18	251.67 ± 69.62	221.0 ± 48.15

**Table 3:** Subgroup analysis of studies reporting ICAM-1 levels in OSA cases compared to controls.

Variable	Subgroup (No. of Studies)	Mean Difference	95%CI		*p*-Value	I^2^
			Lower	Upper		
Ethnicity	Asian (34)	223.53	179.17	267.89	**<0.00001**	100%
	Caucasian (9)	50.66	24.64	76.67	**0.0001**	93%
	Mixed (2)	56.01	−52.34	164.36	0.31	93%
Sample size	≥100 (10)	186.38	103.68	269.08	**<0.00001**	100%
	<100 (35)	181.88	139.61	224.16	**<0.00001**	100%
Mean AHI in cases, events/h	≥30 (21)	180.97	115.92	246.02	**<0.00001**	100%
	<30 (17)	135.54	81.04	190.04	**<0.00001**	99%
Blood sample	Serum (35)	169.29	125.00	213.58	**<0.00001**	100%
	Plasma (10)	238.53	148.42	328.65	**<0.00001**	99%

Bold represents statistical significance (*p* < 0.05).

**Table 4:** Meta-regression analysis of studies reporting ICAM-1 levels in OSA cases compared to controls.

Variable	Coefficient	95% Lower	95% Upper	Z-Value	2-Sided *p*-Value
Publication year	−0.0009	−0.0775	0.0757	−0.02	0.9826
Sample size	0.3228	−0.5487	1.1942	0.73	0.4679
Mean AHI in cases	3.6421	0.5408	6.7433	2.30	**0.0213**

Bold represents statistical significance (*p* < 0.05).

**Table 5:** Results of trim-and-fill method.

Value	Studies Trimmed	Fixed-Effects			Random-Effects			Q Value
		Point Estimate	Lower Limit	Upper Limit	Point Estimate	Lower Limit	Upper Limit	
Observed	-	76.427	74.499	78.355	183.976	143.371	224.579	13,268.783
Adjusted	22	15.741	14.021	17.461	29.675	−14.561	73.912	33,739.560

## Data Availability

Data sharing is not applicable to this article as no datasets were generated or analyzed during the current study.
